# Left ventricular pseudoaneurysm associated with infective endocarditis: an autopsy case report

**DOI:** 10.1186/s41935-022-00294-2

**Published:** 2022-08-19

**Authors:** Razuin Rahimi, Nur Shafaradila Shamsul Anuar, Noor Kaslina Mohd Kornain, Norizal Mohd Noor

**Affiliations:** 1 Department of Forensic Pathology, Faculty of Medicine, Universiti Teknologi MARA, Sungai Buloh Campus, Shah Alam, Selangor Malaysia; 2 Department of Forensic Medicine, Hospital Sungai Buloh, Ministry of Health Malaysia, Sungai Buloh, Malaysia; 3 Department of Pathology, Faculty of Medicine, Universiti Teknologi MARA, Sungai Buloh Campus, Shah Alam, Selangor Malaysia

**Keywords:** Infective endocarditis, Pseudoaneurysm, Valvular disease, Aortic valve, Autopsy

## Abstract

**Background:**

Infective endocarditis (IE) is a bacterial infection of the heart valves or endocardium, with complications such as valve perforation, ring abscess, fistula, or damage to the subaortic structures. This case report aims to illustrate an atypical complication of IE which is a pseudoaneurysm depicting a periannular hemorrhage.

**Case presentation:**

We describe a case of a 19-year-old male youth who presented with fever and cough a few days prior to his demise. There was no known risk factor for IE. The autopsy revealed a bulging anterior surface, upper part of the left ventricle which was soft and slightly fluctuant. Cut section of the heart revealed large vegetations affecting the right and left coronary cusps of the aortic valve. The vegetations at the left coronary cusp were mobile, with necrotic and hollow area underneath, appearing like a cavity and containing blood clots. The course of the cavity was determined to be at the periannular region and contained within the myocardium. These findings were consistent with left ventricular pseudoaneurysm. Culture of the vegetations specimen yielded growth of *Granulicatella adiacens* sp. anti-streptolysin O titre (ASOT) was 400 IU/mL and reported as positive.

**Conclusions:**

IE secondary to *Granulicatella* sp is rare and may result in catastrophic complications. Therefore, this case report is intended to highlight the autopsy findings of the disease as well as to create awareness of its subtle clinical symptoms.

## Background

Infective endocarditis (IE) occurs as a result of bacterial infection of the heart valves or endocardium (Kashyap et al. 2021). Aortic valve endocarditis may cause complications such as valve perforation, ring abscess, fistula, or damage to the subaortic structures (Zencirkıran Ağuş et al. 2018). One of its rare complications is a pseudoaneurysm of the mitral-aortic intervalvular fibrosa (MAIVF). It is also associated with higher mortality rate as it has the tendency to rupture into the pericardium, thus causing a cardiac tamponade (Fazlinezhad et al. 2012). The MAIVF is relatively an avascular structure; therefore, it is prone to infection and injury which resulted in the formation of pseudoaneurysm (Pereira Nunes and Abreu Ferrari 2020; Kuroda et al. 2020). The microorganism associated with this complication is *Staphylococcus aureus (*Silbiger et al. 2013*)*. Other aetiologic agents include nutritionally variant streptococci (NVS) such as *Abiotrophia* and *Granulicatella* which account for about 5–6% of streptococcal endocarditis (Padmaja et al. 2014; Giuliano et al. 2012). Multiple case studies have found that endocarditis caused by *Granulicatella* sp has been associated with higher mortality rate (17%), higher rate for treatment failure (41%), and as high as 27% requirement for surgical intervention (Adam et al. 2015; Vandana et al. 2010). It is due to its fastidious nature that it is frequently associated with negative blood culture, yielding in late bacteriological identification and increased antibiotic resistance to beta-lactam and macrolides (Vandana et al. 2010).

Over the past 2 decades, despite the advancement of diagnostic method and treatment, the mortality rate for IE remains relatively high (Murdoch et al. 2009). A retrospective study done by Fernandez Guerrero et al. reinforced the importance of autopsy in evaluating the quality of care and providing data for the management of IE (Fernandez Guerrero et al. 2012). This case report described autopsy findings of an atypical complication of IE which is a pseudoaneurysm depicting a periannular haemorrhage, in a previously healthy male youth. The causative organism was identified to be *Granulicatella* sp. We also aim to create awareness of its subtle clinical symptoms as common constitutional symptoms of IE were missing in this case.

## Case presentation

A 19-year-old male youth has collapsed at home after complaining of fever and cough for a few days. A day before his demise, his father brought him to a nearby clinic where he was prescribed with fever and cough medications, including antibiotics. Unfortunately, his conditions did not improve, and he was found collapsed in the bathroom. He was immediately brought to a hospital where he was pronounced dead upon arrival at the emergency department. Later, he was brought to the forensic department for a medicolegal autopsy examination. Further history from the family members revealed that he was a healthy teenager with no known medical illness prior to the episode of cough and fever which later led to his premature demise.

**Fig. 1:**
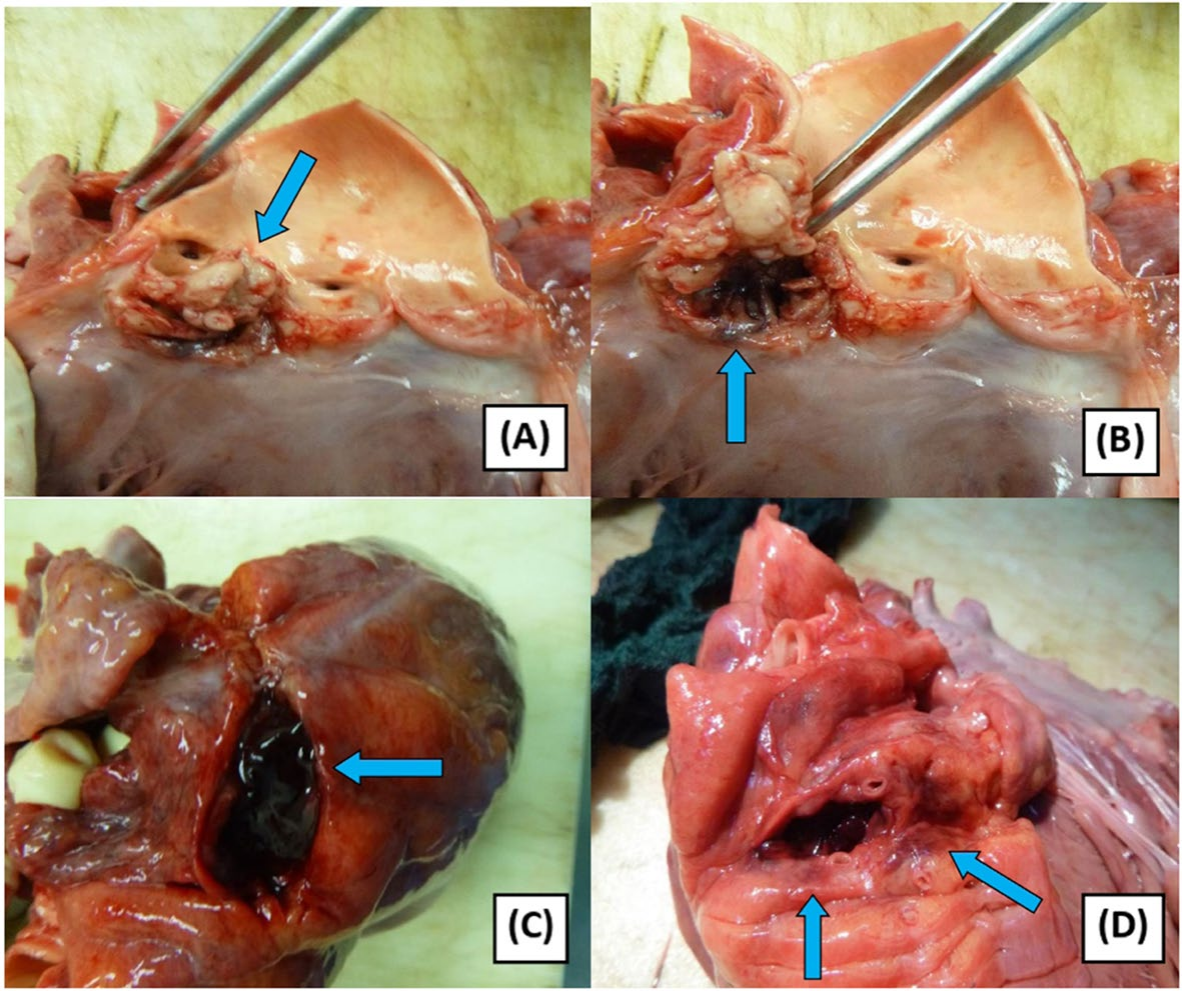
**A** Cut section of the left ventricle of the heart shows a large, mobile vegetations affecting the left coronary cusp of the aortic valve (arrow). The right coronary cusp is slightly affected. **B** There is a hollow area within the myocardium beneath the vegetations, with an ulcerated opening (arrow). **C** Cut section of the bulging epicardial surface of the left ventricle revealed large amount of blood clots within the myocardium (arrow). **D** The pseudoaneurysm contains blood clots and impinges on the coronary artery. The surrounding myocardium is hemorrhagic and soft in consistency, in keeping with acute myocardial infarction (arrows)

Autopsy examination revealed a muscular and large-built male subject, measuring 184 cm in length and 82 kg in weight, with body mass index of 24.2 kg/m^2^. There was no injury or congenital deformity seen. Postmortem changes such as lividity and rigour mortis were present. Slight bluish discolouration of the lips and nail beds was present, indicating cyanosis. The oral cavity showed natural dentition with good oral hygiene. Internal examination of the skull and the brain showed intact skull and normal brain anatomy. Examination of the thoracic organs showed an intact pericardium, with minimal serous effusion. The heart weighed 475 g, denoting cardiomegaly. There was bulging anterior surface, upper part of the left ventricle observed, which was soft and slightly fluctuant on pressure. Cut section of the heart revealed large vegetations appearing like irregular nodular lesions affecting the left and right coronary cusps of the aortic valve (Fig. 1a). The largest vegetation measured 2 cm × 2 cm, completely disrupting the left coronary cusp. The right coronary cusp mainly displayed ulceration, thickening, and small nodules, as a result of direct spread of the lesion from the adjacent cusp. The non-coronary cusp of the aortic valve was sightly affected, exhibiting a small fibrotic nodule. Further examination of the vegetations at the left coronary cusp revealed a mobile lesion with a necrotic and hollow underneath the area, appearing like a cavity, hidden by the large vegetations (Fig. 1b). The cavity which was present within the left ventricular wall measured 3 cm × 2.5 cm × 1 cm, containing blood clots. The course of the cavity was determined to be at the periannular region and contained within the myocardium. It did not rupture to the epicardial surface. Blood clots which were removed from the cavity weighed approximately 10 g. These findings were consistent with left ventricular pseudoaneurysm, a rare complication of infective endocarditis. The coronary arteries were patent. Subendocardial fibrosis was prominent at the left ventricle. The myocardium surrounding the lesion also appeared soft and haemorrhagic, in keeping with acute myocardial infarction. Examination of the other internal organs such as the lungs, liver, spleen, kidneys, and intestines generally showed congestion, with no gross pathology observed.

Several postmortem specimens were obtained for laboratory investigations. Forensic toxicology analysis showed negative results for alcohol and common drugs, from both the blood and urine samples. Rapid test antigen and polymerase chain reaction (PCR) for COVID-19 were negative. Other infectious disease screening also showed negative results for human immunodeficiency virus (HIV) and hepatitis B and C. Blood and lung tissue specimens were sent for microbiology examination as part of a septic work-up in view of the fever and respiratory symptoms. The results of blood culture showed no growth for both aerobic and anaerobic microorganisms. Culture of the vegetations specimen yielded growth of *Granulicatella adiacens* sp. anti-streptolysin O titre (ASOT) was 400 IU/mL and reported as positive.

**Fig. 2:**
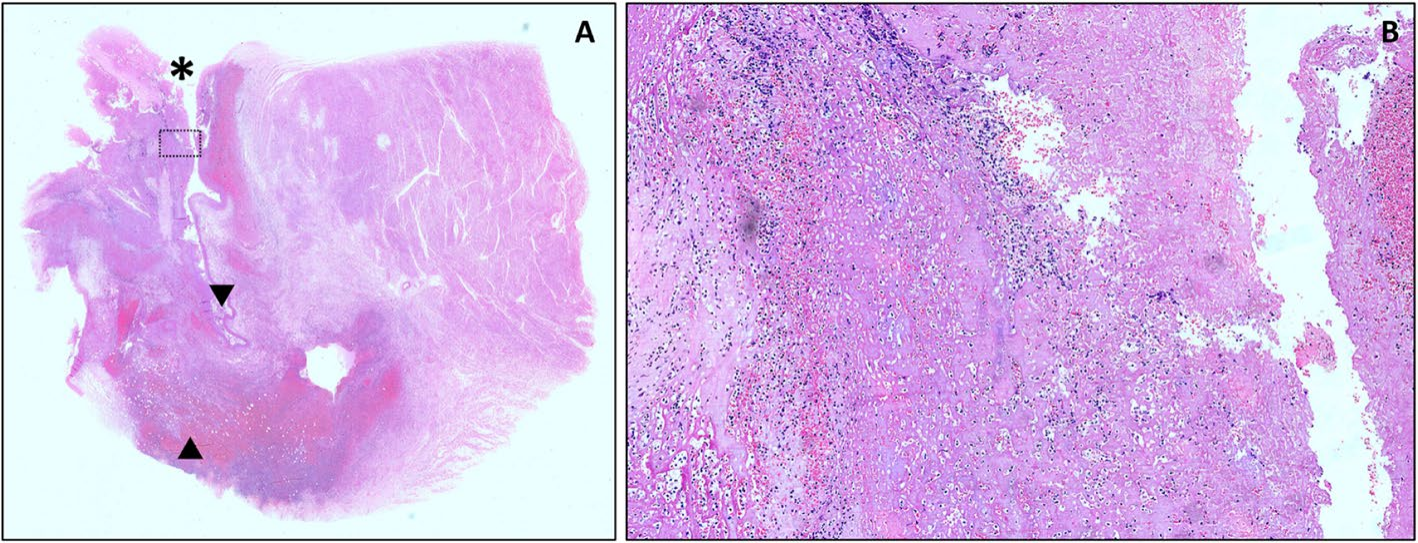
**A** and **B** Section from the cardiac lesion. **A** Low-power view of the lesion, exhibiting the transmural destruction of the myocardial tissue beneath the valve area (*), replaced by granulating fibrous tissue. There is also a formation of pseudoaneurysm (▼) with extensive hemorrhage (▲) that represents the rupture site. **A** High-power view shows the fibrin vegetation deposits that are seen at the valve and wall of the pseudoaneurysm, extensively infiltrated by neutrophils

Representative tissue samples from the cardiac lesions were obtained for histology and stained with haematoxylin and eosin (H&E) stains. Microscopically, there are areas of complete destruction of the myocardial layer of the heart wall, beneath the valve. These are covered by fibrofatty tissue that undergoes extensive granulation, haemorrhage with acute and chronic inflammatory cell infiltrations. It subsequently forms a tamponade and therefore architecturally constitutes a formation of a pseudoaneurysm. An extensive haemorrhage is seen in one area of the pseudoaneurysm wall, indicating a rupture. The attached valve tissues are mainly fibrotic and myxomatous with many areas showing necrosis. It is mainly covered by fibrin deposits, infiltrated by mostly acute inflammatory cells with some forming exudates. There fibrin deposits or vegetations are present continuously throughout the internal surface of the pseudoaneurysm lining, which are also mainly infiltrated by neutrophils (Fig. 2). Special stains performed on the sections, namely Ziehl-Neelsen for acid fast bacilli, Grocott’s methenamine silver stain for fungal bodies, and Giemsa and Gram stains for bacterial colonies, show no specific infective agent present.

Correlating the autopsy findings and the laboratory investigation results, the cause of death was certified as aortic valve infective endocarditis complicated with periannular haemorrhage, secondary to pseudoaneurysm.

## Discussion

IE is known to have high morbidity and mortality rates, regardless of age group. In young population, risk factors for IE include congenital as well as acquired valve diseases. In North America, an important risk factor includes intravenous drug use, as users from this category contributed 16% of the IE incidence. Injection drug use may lead to IE through direct injection of microorganisms from the skin and soft tissue into the bloodstream. A study has found that 5 to 20% of injection drug users have had IE (Murdoch et al. 2009; Wurcel et al. 2016). Other risk factors include prosthetic heart valves, dental caries, and procedures (Kashyap et al. 2021). In this case report, the deceased succumbed to death after having fever and cough for a few days. He was a healthy young man with no known risk factors. He also did not show any cardiovascular symptoms prior to death. An almost similar case was reported by Kashyap et al., where severe IE was diagnosed in a healthy young man with no known risk factor and presented with left-sided abdominal pain, fever, and vomiting. Fortunately, prompt and accurate diagnosis was made, and he had a full recovery following antibiotics administration and aortic valve replacement surgery (Kashyap et al. 2021).

The infective organism detected in this case is *Granulicatella adiacens* sp. It is a normal flora of human mouth as well as intestinal tracts, belonged to NVS of the viridans group (Padmaja et al. 2014; Adam et al. 2015). Although infections due to NVS are quite uncommon, the microorganism has been implicated in other types of infection such as central nervous system and ocular and respiratory tract infections (Padmaja et al. 2014). IE caused by this microorganism often causes subacute or chronic presentation. While it is rare, it is generally associated with severe condition (Padmaja et al. 2014; Adam et al. 2015). A case series reported by Adam et al. showed 4 cases of IE involving this microorganism, with 11% of the cases had complications of perivalvular abscess. In the case series, the mortality rate was 17% (Adam et al. 2015). In all these patients, risk factors such as poor oral hygiene, dental extraction, and heart valve prosthesis were identified. Constitutional clinical symptoms such as weight loss, anorexia, and fever were present for a few months before heart failure and embolism ensued (Padmaja et al. 2014; Adam et al. 2015). In this case report, as we could not establish a risk factor, the portal of entry of the microorganism into the bloodstream was not known. However, it was assumed to have originated from the oral cavity. We also hypothesized that the deceased probably had very subtle and nonspecific clinical symptoms prior to this final episode; thus, it did not alert the parents to seek an appropriate medical treatment.

A variety of complications may result from IE. Cardiac structural complications may occur when the infection spreads within the heart, giving rise to pseudoaneurysm or periannular abscess respectively (Mocchegiani and Nataloni 2009). While these complications are rare, they also carry higher mortality rate if conditions are not recognized early and treated promptly (Bhimani et al. 2020). Pseudoaneurysm formation is believed to develop from extravalvular abscesses undergoing remodelling. As it is always seen to have a communication with a high-pressure chamber such as the left ventricle or the aorta, it has been suggested to develop from a previously weakened abscess undergoing necrosis (Silbiger et al. 2013). The high pressure within the left ventricle is believed to dissect through the abscess into the underneath structure.

The mechanism also explains the sac-like expansion of the pseudoaneurysm (Silbiger et al. 2013). In the left ventricle, it usually occurs at the anterior wall, as it commonly develops from abscesses in the MAIVF. The pseudoaneurysm decompressed by forming a fistula with the left atrium or perforate the epicardium and cause bleeding into the pericardial sac, giving rise to cardiac tamponade and immediate death. The size, location, and extent of the structural anomaly too may cause compression of the coronary artery causing acute myocardial infarction (Fazlinezhad et al. 2012; Silbiger et al. 2013). As in this case, we postulate that the damage made by the infective material weakens the myocardium and allows dissection to occur. The appearance of the opening of the pseudoaneurysm which resembles a slit like or dissection into the myocardium further affirmed the theory of extravalvular abscess remodelling. Thus, the main content of the pseudoaneurysm is mainly thrombus with minimal infective material. This is also proven histologically, as it shows many areas of fibrin deposition within the myocardium with minimal neutrophilic infiltrations (Fig. 2).

## Conclusions

IE secondary to *Granulicatella* sp. is rare and may result in catastrophic complications. Therefore, a case report highlighting such an uncommon complication of a common disease proved to be of an utmost importance. In this report, not only the authors wished to illustrate the remarkable autopsy findings of the disease but also we wished to create awareness of its subtle clinical symptoms.

## Data Availability

All relevant data supporting the conclusions of this article are included within the article.

## Abbreviations

IE: Infective endocarditis
ASOT: Anti-streptolysin O titre
MAIVF: Mitral-aortic intervalvular fibrosa
NVS: Nutritionally variant streptococci
PCR: Polymerase chain reaction
HIV: Human immunodeficiency virus
H&E: Haematoxylin and eosin stain
